# Can endophytic microbial compositions in cane roots be shaped by different propagation methods

**DOI:** 10.1371/journal.pone.0290167

**Published:** 2023-08-15

**Authors:** Da Yang, Xinru Lin, Yufei Wei, Zujian Li, Haodong Zhang, Tian Liang, Shangdong Yang, Hongwei Tan

**Affiliations:** 1 Guangxi Key Laboratory of Agro-Environment and Agro-Products Safety, National Demonstration Center for Experimental Plant Science Education, Agricultural College, Guangxi University, Nanning, China; 2 Guangxi Key Laboratory of Sugarcane Genetic Improvement, Guangxi Academy of Agricultural Sciences, Nanning, China; Nuclear Science and Technology Research Institute, ISLAMIC REPUBLIC OF IRAN

## Abstract

In practical production, cane stems with buds are generally used as seed for propagation. However, long-terms cane stems only easily lead to some problems such as disease sensitivity, quality loss, etc. Recently, cane seedings, which are produced by tissue culture were used in sugarcane production, but few studies on cane health related to tissue culture seedings. Therefore, to evaluate the immunity and health of sugarcanes growing from different reproduction modes, the endophytic microbial compositions in cane roots between stem and tissue culture seedlings were analyzed using high-throughput techniques. The results showed that the endophytic microbial compositions in cane roots were significant differences between stem and tissue culture seedlings. At the genus level, *Pantoea*, *Bacillus*, *Streptomyces*, *Lechevalieria*, *Pseudomonas*, *Nocardioides*, *unclassified_f__Comamonadaceae* enriched as the dominant endophytic bacterial genera, and *Rhizoctonia*, *Sarocladium*, *Scytalidium*, *Wongia*, *Fusarium*, *unclassified_f__Phaeosphaer*, *unclassified_c__Sordariom*, *unclassified_f__Stachybot*, *Poaceascoma*, *Microdochium*, *Arnium*, *Echria*, *Mycena* and *Exophiala* enriched as the dominant endophytic fungal genera in cane roots growing from the tissue culture seedlings. In contrast, *Mycobacterium*, *Massilia*, *Ralstonia*, *unclassified_f__Pseudonocardiacea*, *norank_f__Micropepsaceae*, *Leptothrix* and *Bryobacter* were the dominant endophytic bacterial genera, and *unclassified_k__Fungi*, *unclassified_f__Marasmiaceae*, *Talaromyces*, *unclassified_c__Sordariomycetes* and *Trichocladium* were the dominant endophytic fungal genera in cane roots growing from stem seedlings. Additionally, the numbers of bacterial and fungal operational taxonomic units (OTUs) in cane roots growing from tissue culture seedlings were significantly higher than those of stem seedlings. It indicates that not only the endophytic microbial compositions in cane roots can be shaped by different propagation methods, but also the stress resistance of sugarcanes can be improved by the tissue culture propagation method.

## Introduction

Sugarcane (*Saccharum* spp.) is a monocotyledonous herbaceous plant belonging to the grass family, and it is a typical tropical and subtropical crop [1]. Sugarcane is a global crop used as a material for sugar, biofuel and electricity production [2]. In practice, sugarcane is usually propagated with vegetative method [3]. i.e., sugarcane propagation and cultivation have been carried out with two or three seed stems in production [4,5]. However, seed stems also make quite a lot of problems in sugarcane production. For example, root stem easily induces shoot damage due to pathogen invasion or environmental stresses, such as drought, high and low temperatures, waterlogging, etc. At present, an alternative propagation method for sugarcane production is urgently needed [6]. The stem seedlings have a disadvantage, for they are responsible for the transmission of various pathogens that accumulate in the plant during the cultivation cycle [7].

Till now, plant tissue culture, also known as micropropagation has become a common technique in use for genetic improvement programs [8,9]. Tissue culture can be used in avoiding the adverse impact of environmental factors on plants [10,11]. Meanwhile, plenty of virus-free sugarcane seedlings also can be propagated within a short time by tissue culture method which compares to traditional methods [12,13]. Tissue culture technology can offer the assurance of plant material free from diseases and pest infestations in destructive to conventional plantations [14]. For example, sugarcane black spike disease, yellows virus and yellows phytoplasma can be eliminated by tissue culture [15,16]. Cane yield and quality also can be increased by the use of tissue seedlings [17] The plant tissue culture technique has been proven to be useful in maintaining gene pools in vitro and it is also a means of clonal propagation [18].

Recently, advances in the microbiome have revealed a lot of perceptions in plant microbial interactions and opened up a new field in microbiome-derived biotechnologies for application in agricultural and industrial productions [19,20]. Endophytic microorganisms, mainly bacteria and fungi, inhabit the interior of the host plants for one period or their life cycle without inducing disease symptoms or producing external structures [21]. Nowadays, the interior of plants is considered as a prolific environment for the discovery of endophytic microorganisms with new biological activities, particularly, the biocontrol capabilities are the urgent tasks [22,23]. Endophytes play an important role in maintaining host health as they confer tolerance/resistance to abiotic and biotic stresses in host plants as well as increase plant growth and crop yield [24–26]. Meanwhile, endophytic bacteria can ensure the nutrient intake of the plant by enhancing the uptake of mineral elements such as nitrogen [27,28], phosphorus solubilization, nitrogen fixation and potassium uncoupling, etc., iron-producing carriers and secretion of probiotic phytohormones (such as indole 3-acetic acid (IAA), gibberellin, and cytokinin, etc.) [29]. Additionally, endophytic bacteria also can effectively regulate defense-related signaling pathways in the host and achieve self-colonization by establishing symbiotic associations [30].

Therefore, to evaluate cane health and the immune capacity growing from different propagation methods, the endophytic microbial community structures in cane roots between stem and tissue culture seedlings were analyzed.

## Material and methods

### Field site description and treatment

The trial site was located at the sugarcane farm in Fusui County, Guangxi Zhuang Autonomous Region (107°55′4″E, 22°44′56″N). The sugarcane variety was Zhongtang 3, which was planted in March 2022. The physio-chemical properties of the soil were as follows: pH 5.42, organic matter content 11.26 g·kg^-1^, total nitrogen, phosphorus and potassium 0.68 g·kg^-1^, 0.75 g·kg^-1^, 12.64 g·kg^-1^, respectively. Meanwhile, the available nitrogen, phosphorus and potassium were 20.78 mg·kg^-1^, 0.57 g·kg^-1^, and 108.67 g·kg^-1^, respectively.

There were two treatments: TC treatment: sugarcane tissue culture propagation; CS treatment: sugarcane conventional seedlings propagation. Each treatment was replicated three times and arranged in randomized groups, with a total of 6 plots (100 × 100 m each). Irrigation and pest control were carried out by conventional methods.

Sugarcane axillary buds were used as explants. Firstly, were first washed with 0.1% mercuric chloride solution and then were washed in sterile distilled water at least three times before using them for tissue culture experiments. Secondly, shoots were placed on a standard shoot medium consisting of basal MS medium (Murashige and Skoog 15) supplemented with 1962.2 mg/L 5-BA (6-benzylaminopurine). For proliferation, the same medium was used along with 1.0 mg/L 6-BA and 0.5 mg/L KT (kinetin) and 3.0 mg/L NAA (1-naphthaleneacetic acid). Meanwhile, 30 g/L sugar and 4.0 g/L agar were added to all medium formulations and the pH was adjusted to 5.8. A light intensity of 12 lx was set at 25°C with a photoperiod for shoot or seedling growth. When shoots reached 30 ± 5 mm in length, they were then passaged and cultured for 20 days. Subsequently, 25–30 mm long seedlings were transferred to the rooting medium for 25 d and then subsequently colonized in fields [31].

In addition, 90,000 buds per ha approximately were sown and 300 kg·ha^−2^ of urea, 75 kg·ha^−2^ of K_2_O, and 300 kg·ha^−2^ of calcium superphosphate were applied to all plots. Moreover, top dressings with 30% and 70% of the total fertilizer application at seedings and cane elongation periods, respectively [32].

### Root samples collection

Sugarcane plant samples were randomly collected from the sugarcane cultivation area on November 29, 2022. Three sugarcane plants with uniform growth were chosen at random. The plants were then dug up with the stem at the center to create a loose, circular inter-root circle, and the entire plant was then pulled up with soil while being held at the base and the soil attached to the roots was shaken off [33]. Bring back to the laboratory. Plant samples were rinsed with sterile water and the sterile filter paper was used to remove surface soil and appendages.

All samples were packed into marked sealed sterile bags and immediately transferred to the laboratory, stored in a -80°C refrigerator for later analysis.

### Soil chemical and biological properties

Soil pH was measured using a pH meter (soil: water ratio 1:2.5). Organic matter content was determined using the potassium dichromate-sulfate colorimetric method [34]. Total nitrogen, phosphorus and potassium contents were determined using the semimicro-Kjeldahl [35], alkali fusion-molybdenum anti-colorimetric and alkali fusion-flame spectrophotometry [36], respectively. Meanwhile, the available P, N, and K contents were also determined by acid-fluoride solutions [37], alkali diffusion and flame photometry methods [36], respectively.

### Endophytic microbial composition analysis

The extraction, PCR amplification and sequencing of total DNA from root samples were completed by Shanghai Mayobio Biomedicine Technology Co., Ltd., China.

The E.Z.N.A. DNA Kit (Omega Company, Norwalk, CT, USA) was used to extract total DNA. A NanoDrop 2000 spectrophotometer (Thermo Company, Waltham, NJ, USA) was used to measure the concentration and purity of the DNA, and a 1% agositol gel was used to verify the purity and quality of the genomic DNA. Using root endophytic bacterial DNA as a template, the V5-V7 hypervariable region of the bacterial 16S rRNA gene was amplified using the primers 799F (5′-AACMGGATTAGATACCCKG-3) and 1193R (5′-ACGTCATCCCCACCTTCC-3′). Additionally, utilizing the DNA of endophytic fungi as a template, ITS1F(5’-CTTGGTCATTTAGAGGAAGTAA-3’) and ITS2R(5’-GCTGCGTTCTTCATCGATGC-3’) primers were created. Products were recovered by 2% agarose gel electrophoresis, purified by AxyPrep DNA Gel Recovery Kit (AXYGEN) (Axygen Biosciences, Union City, CA, USA), eluted by Tris HCl, and quantified with QuantiFluor-STTM after CR utilizing ABI GeneAmptype 9700 (ABI, Carlsbad, CA, USA). Purified amplicons are constructed into libraries according to the standard operating procedures of the Illumina MiSeq platform. Illumina’s MiSeq PE300 platforms were used for sequencing.

Processing of sequencing data. According to the following standards, the raw 16S rRNA gene-sequencing reads were demultiplexed, quality-filtered with fastp version 0.20.0 [38] and combined with FLASH version 1.2.7 [39]; (i) Reads containing ambiguous characters were also removed, and the 300 bp reads were shortened at any site with an average quality score of 20 over a 50 bp sliding window; (ii) Only overlapping sequences longer than 10 bp were put together in accordance with their overlapped sequence; the maximum mismatch ratio of overlapped areas was 0.2; and (iii) samples were diluted before analysis [40].

UPARSE (version 7.1, http://drive5.com/uparse/) was used to cluster operational taxonomic units (OTUs) with a 97% similarity cutoff, and chimeric sequences were found and eliminated [41,42]. Using a confidence threshold of 0.7 and the 16S and ITS rRNA database, RDP Classifier (http://rdp.cme.msu.edu/) version 2.2 was used to assess the taxonomy of each OTU representative sequence [43].

The LEfSe analysis’s LDA score was set to 3.5, and the Wilcoxon rank sum test was performed to see whether there were any differences between the groups. Additionally, the LDA Score was utilized to analyze and lessen the impact of species with substantial differences.

PICRUSt was used to remove the effect of the number of copies of the 16S marker gene in the genome of the species and to standardize the OTUs abundance table, using the green gene ID corresponding to each OTUs. Each OTU’s matching KEGG Orthology (KO) information and COG family information were acquired, and the abundance of each COG and KO could then be computed. The functional and descriptive data for each COG were obtained by parsing the COG database against the eggNOG database [44].

The FunGuild annotation tool was used to identify the different functional groups in the fungal community, categorizing the fungal taxa into three trophic modalities–saprotrophy, symbiotrophy and pathotrophy. These modes were further subdivided into specific guilds comprised of fungi that share similar lifestyle modes [45].

### Statistical analyses

Online date on Majorbio Cloud Platform (http://www.majorbio.com) of the Majorbio Bio-pharm Technology Co. Ltd. (Shanghai, China) was analyzed. *Alpha* diversities of bacterial and fungal communities were calculated using Mothur (version v.1.30.2, https://mothur.org/wiki/calculators/). Linear discriminant analysis (LDA) was performed using LEfSe analysis. Excel 2019 and IBM SPSS Statistics 26 (IBM Crop., Armonk, New York, NY, USA) were used to evaluate the experimental data. And the mean values of microbial biomass, endophytic bacterial and fungal diversities and richness were compared by Student’s t-test using Statistical Products and Service Solutions (SPSS version 26) software with a significance level of 0.05. Also, the data are presented as means and standard deviations (mean ± SD).

## Results

### Diversity of endophytic microbial communities in sugarcane roots growing from different reproduction modes

As shown in Table 1, the coverage of all samples was 99%, they indicated that the data were reliable. First, the endophytic bacterial diversity (Shannon and Simpson indices) and richness (Ace and Chao1) indices in sugarcane roots growing from tissue culture (TC) and Conventional Seedlings (CS) were all not significantly different from each other. In addition, endophytic fungal diversity and richness between TC and CS treatments were also shown the same trends with endophytic bacterial diversity and richness.

**Table 1:** Diversity of endophytic microbial community in cane roots growing from different propagation methods.

	Treatments	Shannon	Simpson	Ace	Chao1	coverage
bacteria	TC	3.83±1.17a	0.10±0.12a	682.50±76.86a	684.11±67.49a	0.99
	CS	4.02±0.17a	0.06±0.02a	677.46±21.76a	686.20±48.85a	0.99
fungi	TC	2.39±0.28a	0.16±0.04a	211.71±54.59a	208.28±50.32a	0.99
	CS	1.19±0.88a	0.56±0.31a	214.97±5.54a	180.09±24.23a	0.99

Note: All data are presented as the mean ± SD (standard deviation). Significant variations between treatments at *P* < 0.05 are indicated by different letters in the same column. TC: Tissue culture; CS: Conventional Seedlings.

### Endophytic microbial community compositions

#### Endophytic bacteria

**Fig 1:**
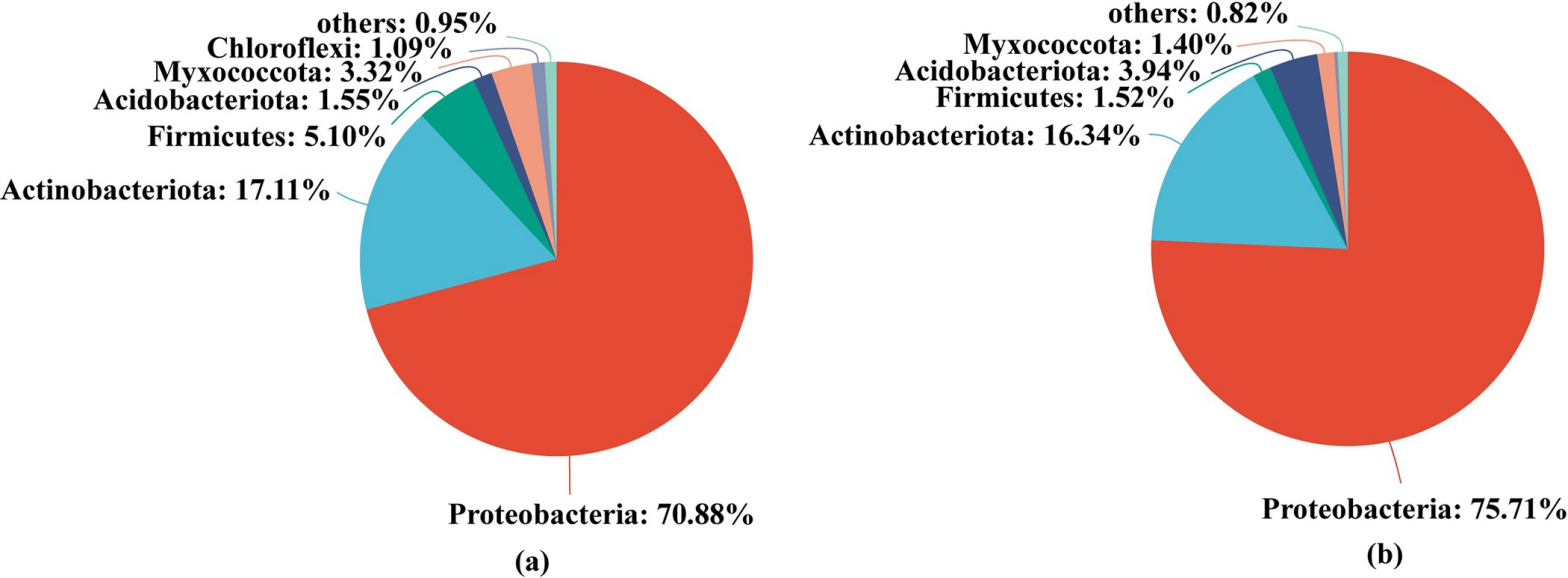
Two different ways of bacterial phylum levels. (a) level of bacterial phylum of sugarcane roots propagated by tissue culture; (b) level of bacterial phylum of sugarcane roots propagated by traditional propagation.

Firstly, at the phylum level, the numbers of dominant endophytic bacterial phyla (the proportions are greater than 1%) in sugarcane roots between tissue culture (TC) and conventional seedlings (CS) were 6 and 5, respectively. Meanwhile, Proteobacteria, Actinobacteriota, Firmicutes, Acidobacteriota and Myxococcota were the common dominant endophytic bacterial phyla in sugarcane roots between TC and CS treatments; However, Chloroflexi was only detected in cane roots of TC treatment (Fig 1A and 1B).

Besides, at the genus level, the numbers of dominant bacterial genera (proportions are greater than 1%) in sugarcane roots growing from TC and CS treatments were 16 and 17, respectively.

**Fig 2:**
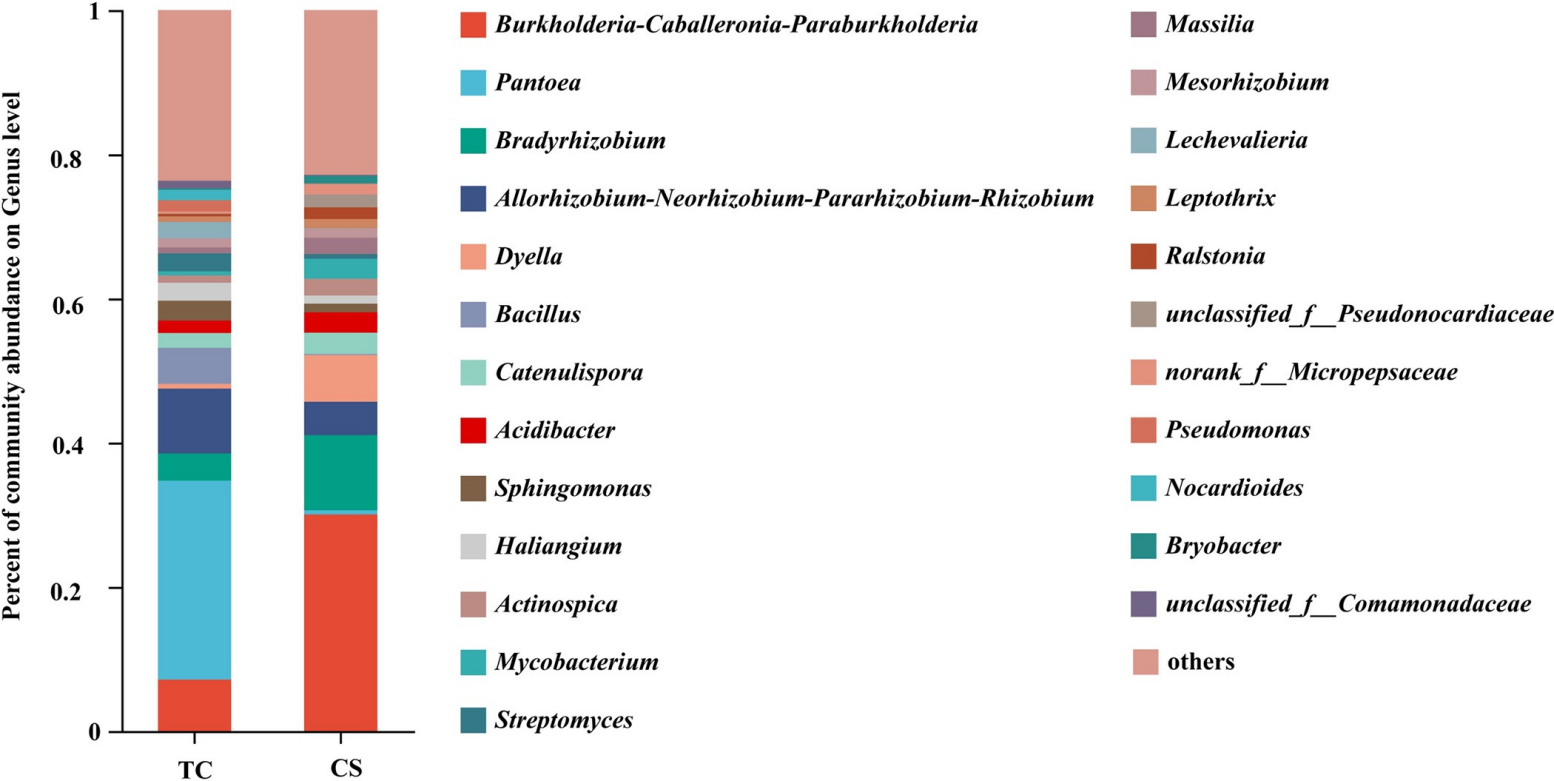
Level of endophytic bacterial genera in cane roots by tissue culture propagation method.

The proportions of dominant endophytic bacterial genera in cane roots growing from TC from high to low were *Pantoea* (27.60%), *Allorhizobium-Neorhizobium-Pararhizobium-Rhizobium* (9.00%), *Burkholderia-Caballeronia-Paraburkholderia* (7.14%), *Bacillus*(5.00%), *Bradyrhizobium* (3.76%), *Sphingomonas* (2.76%), *Haliangium* (2.48%), *Streptomyces* (2.48%), *Lechevalieria* (2.26%), *Catenulispora* (2.04%), *Acidibacter* (1.73%), *Pseudomonas* (1.56%), *Nocardioides* (1.45%), *Mesorhizobium* (1.26%), *unclassified_f__Comamonadaceae* (1.07%), *Actinospica* (1.03%), respectively (Fig 2).

In contrast, the proportions of dominant endophytic bacterial genera in cane roots growing from CS from high to low were *Burkholderia-Caballeronia-Paraburkholderia*(30.04%)、*Bradyrhizobium* (10.41%), *Dyella* (6.48%), *Allorhizobium-Neorhizobium-Pararhizobium-Rhizobium* (4.63%), *Catenulispora* (2.97%), *Acidibacter* (2.82%), *Mycobacterium* (2.77%), *Actinospica* (2.33%), *Massilia* (2.26%), *unclassified_f__Pseudonocardiacea* (1.79%), *Ralstonia* (1.62%), *norank_f__Micropepsaceae* (1.44%), *Leptothrix* (1.30%), *Mesorhizobium* (1.27%), *Sphingomonas* (1.21%), *Haliangium* (1.11%), *Bryobacter* (1.07%), respectively (Fig 2).

Meanwhile, *Pantoea*, *Streptomyces*, *Lechevalieria*, *Pseudomonas*, *Nocardioides*, *unclassified_f__Comamonadaceae* were the specific dominant endophytic bacterial genera in cane roots growing from TC treatment; By contrast, the unique dominant endophytic bacterial genera in the CS treatment were *Mycobacterium*, *Massilia*, *unclassified_f__Pseudonocardiacea*, *Ralstonia*, *norank_f__Micropepsaceae*, *Leptothrix*, *Bryobacter*.

**Fig 3:**
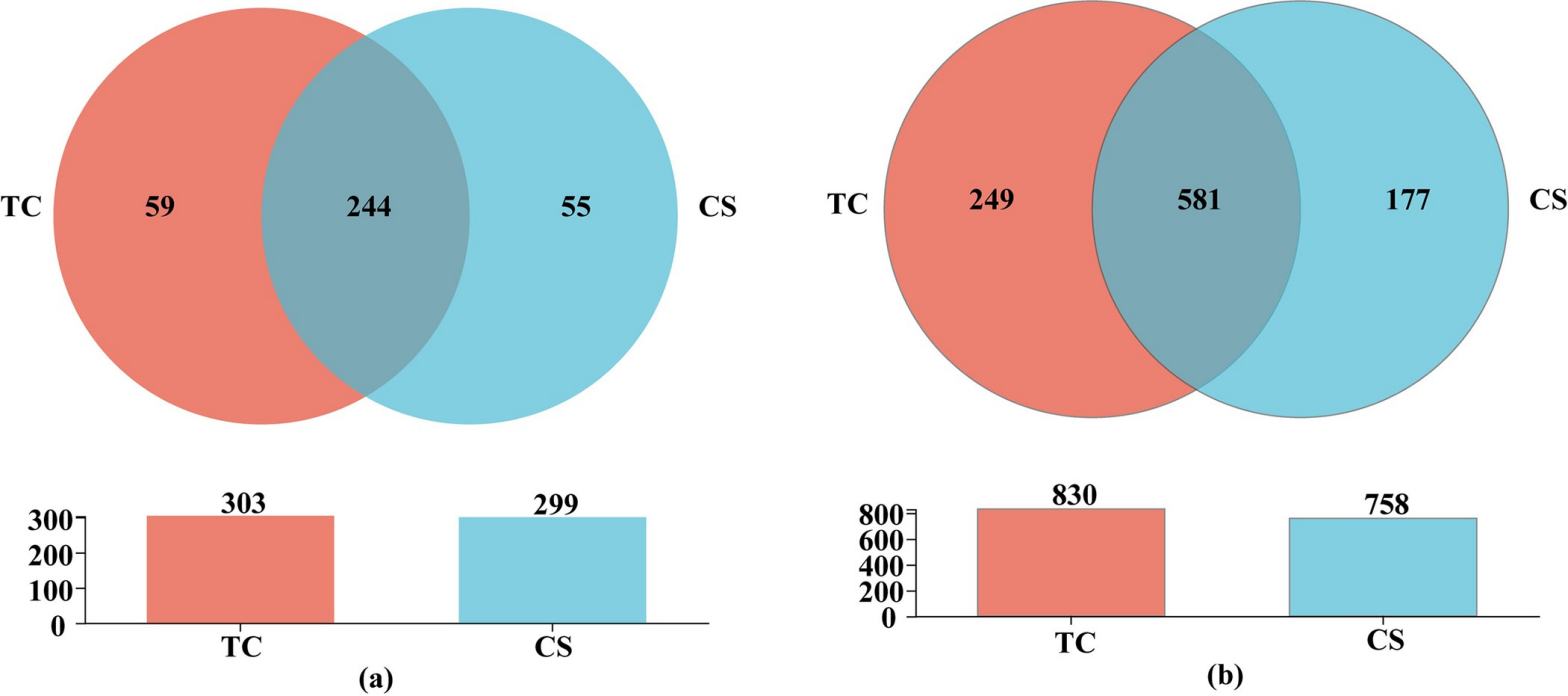
Venn diagram analysis of bacteria in cane roots of two propagation methods. (a) Venn diagram analysis of bacteria at the genus level; (b) Venn diagram analysis of bacteria at the OTUs level.

As shown in Fig 3, 303 and 299 endophytic bacterial genera could be found between tissue culture and conventional seedlings, respectively. Meanwhile, the numbers of unique endophytic bacterial genera were 59 and 55, respectively (Fig 3A). In addition, 830 and 758 endophytic bacterial OTUs could be detected in sugarcane roots growing from tissue culture and conventional seedlings, respectively. In addition, 249 and 177 unique endophytic bacterial OTUs were found in cane roots of tissue culture and conventional seedlings, respectively (Fig 3B).

### Endophytic fungi

**Fig 4:**
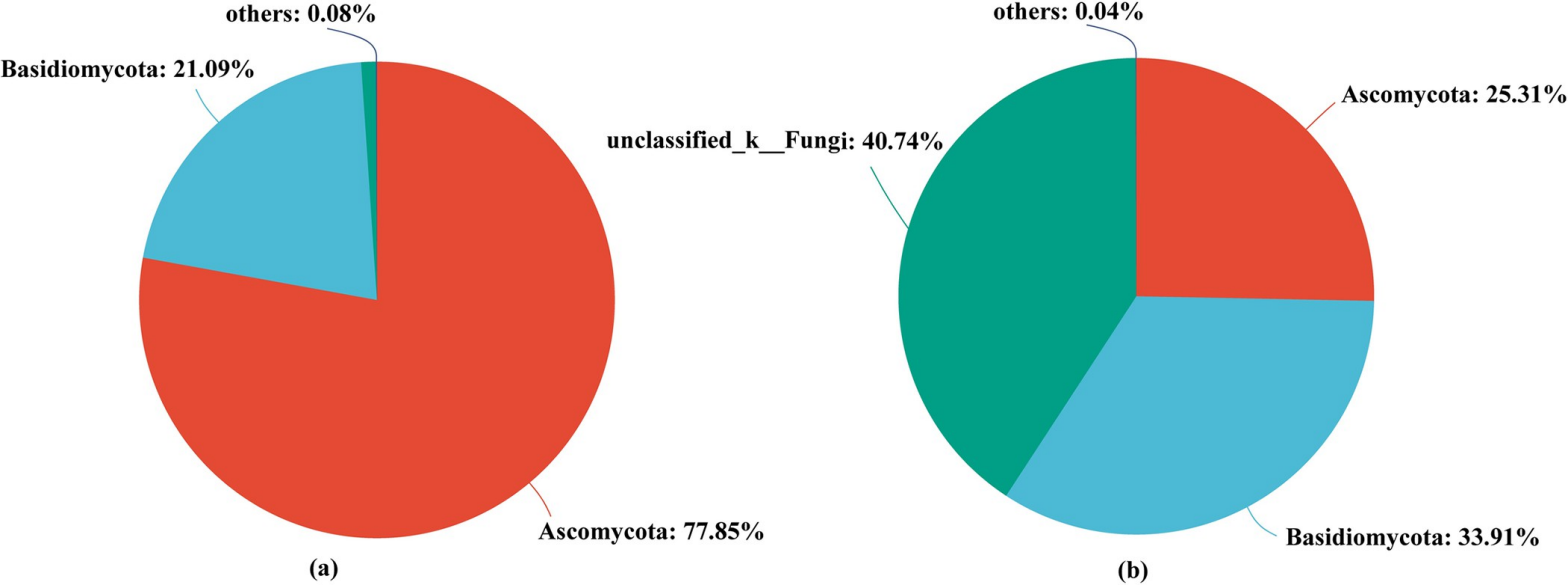
Two different propagation methods of fungal phylum levels. (a) level of fungal phylum in cane roots growing from tissue culture; (b) level of fungal phylum in cane roots growing from conventional propagation methods (stem seedlings).

At the phylum level, the numbers of dominant fungal phyla in cane roots growing from TC and CS seedlings were 2 and 3, respectively. Among them, Ascomycota and Basidiomycota were the common dominant endophytic fungal phyla in cane roots growing from TC and CS seedlings. However, unclassified_k__Fungi was the specific fungal phylum in cane roots growing from conventional seedlings. Meanwhile, the relative abundance of Ascomycota in cane roots growing from tissue culture seedlings was higher than that of conventional seedlings (Fig 4A and 4B).

**Fig 5:**
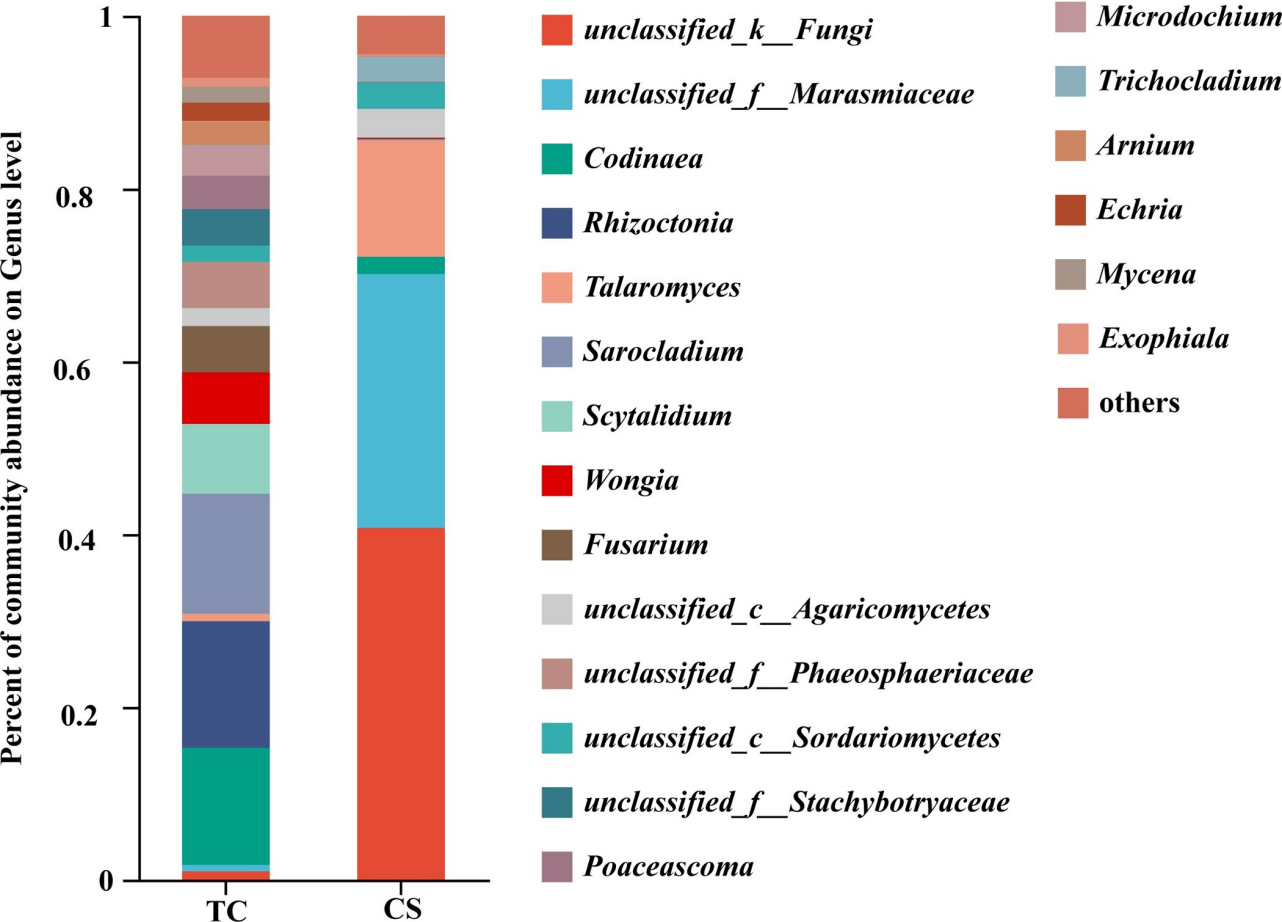
Level of endophytic fungal genera in cane roots by different propagation methods.

In addition, at the genus level, the numbers of dominant endophytic fungal genera (the proportions are greater than 1%) in cane roots growing from tissue culture and conventional seedlings were 16 and 7, respectively (Fig 5).

The proportions of dominant endophytic fungal genera in cane roots growing from tissue culture seedlings from high to low were *Rhizoctonia* (14.63%), *Sarocladium* (13.88%), Codinaea (13.57%), *Scytalidium (8*.*09%)*, *Wongia* (5.96%), *unclassified_f__Phaeosphaer* (5.36%), *Fusarium* (5.26%), *unclassified_f__Stachybot* (4.02%), Poaceascoma (3.88%), Microdochium (3.42%), Arnium (2.82%), *unclassified_c__Agaricomycetes* (2.09%), *Echria* (2.08%), *unclassified_c__Sordariom* (1.88%), *Mycena* (1.86%), *Exophiala* (1.02%), respectively.

In contrast, the proportions of dominant endophytic fungal genera in cane roots growing from conventional seedlings from high to low were *unclassified_k__Fungi* (40.74%), *unclassified_f__Marasmiaceae* (29.38%), *Talaromyces* (13.48), *unclassified_c__Agaricomycetes* (3.03%), *unclassified_c__Sordariomycetes* (3.03), *Trichocladium* (2.92), *Codinaea* (2.00%), respectively.

Among them, *Rhizoctonia*, *Sarocladium*, *Scytalidium*, *Wongia*, *unclassified_f__Phaeosphaer*, *Fusarium*, *unclassified_f__Stachybot*, Poaceascoma, Microdochium, Arnium, *Echria*, *unclassified_c__Sordariom*, *Mycena*, *Exophiala* were the specific dominant endophytic fungal genera in sugarcane roots growing from tissue culture seedlings; By contrast, *unclassified_k__Fungi*, *unclassified_f__Marasmiaceae*, *Talaromyces*, *unclassified_c__Sordariomycetes*, *Trichocladium* were the unique dominant endophytic fungal genera in cane roots of conventional seedlings.

**Fig 6:**
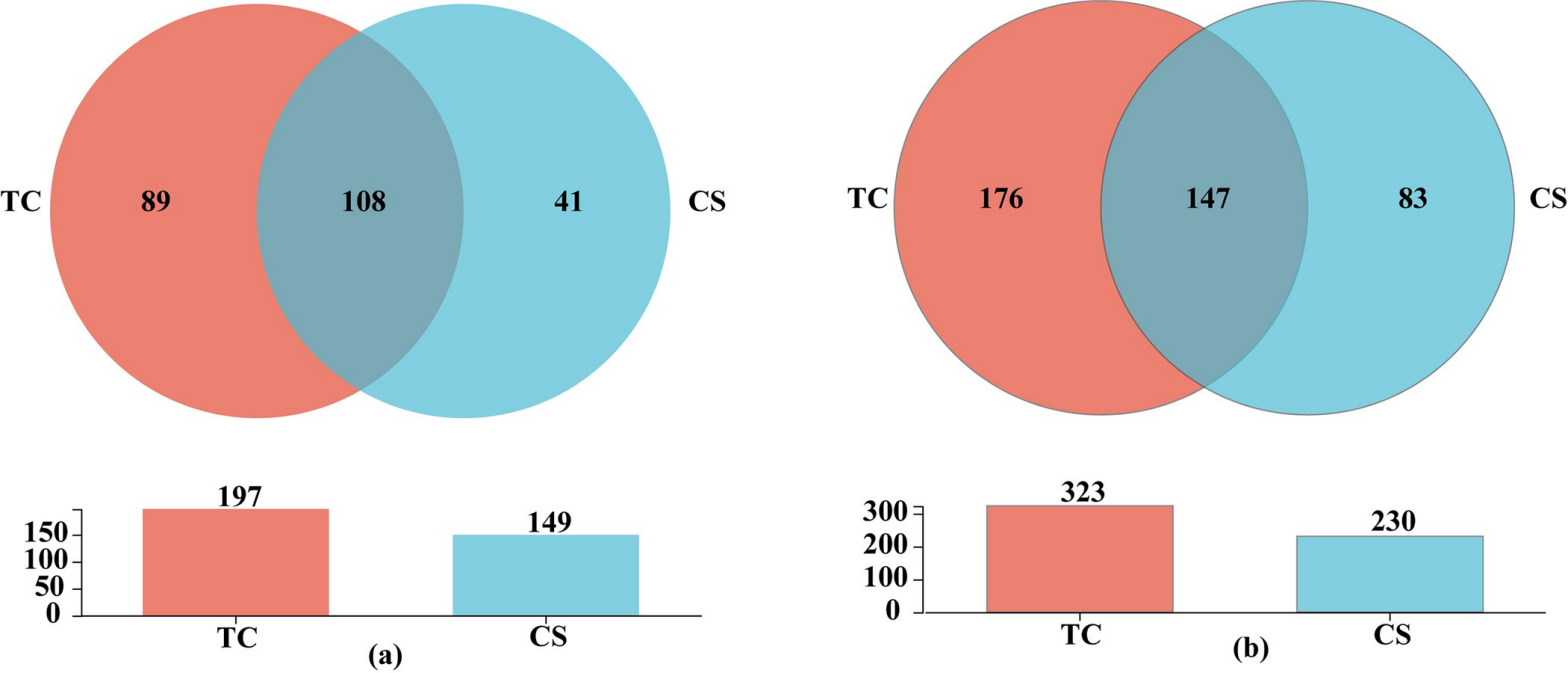
Venn diagram analysis of endophytic fungi in cane roots of two propagation methods. (a) Venn diagram analysis of the endophytic fungi at the genus level; (b) Venn diagram analysis of the endophytic fungi at the OTUs level.

At the genus level (Fig 6A), 197 and 149 endophytic fungal genera could be detected in cane roots between tissue culture and conventional seedlings, respectively. Among them, 89 and 41 specific fungal genera in cane roots of the tissue culture and conventional seedlings, respectively. Furthermore, 323 and 230 fungal OTUs in cane roots of the tissue culture and conventional seedlings, respectively. And 176 and 83 unique endophytic fungal OTUs could be found in cane roots of tissue culture and conventional seedlings at the OTUs level, respectively (Fig 6B).

The above results showed that the numbers of endophytic fungal genera and OTUs were all significantly higher in cane roots growing from tissue culture seedlings than those of conventional seedlings.

### The LEfSe analysis

**Fig 7:**
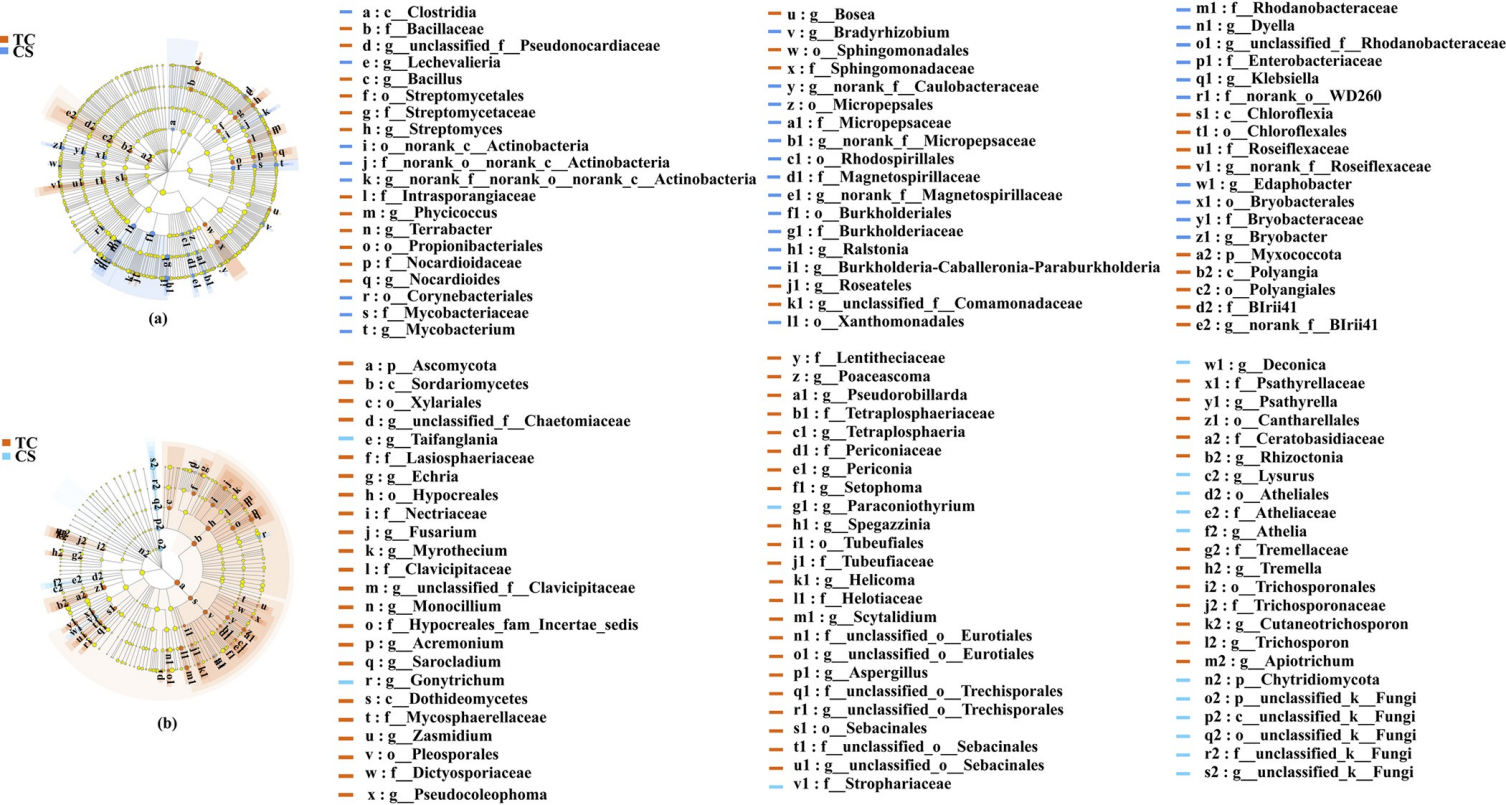
LEfSe analysis of endophytes in cane roots growing from two different propagation methods. (a) bacterial LEfSe analysis; (b) fungal LEfSe analysis. (*P* < 0.05, LDA score  =  3.5).

The LEfSe analysis was also carried out to identify the definitive values of endophytes in cane roots growing from tissue culture (TC) and conventional seedlings (CS). As shown in Fig 7A, a total of 57 endophytic bacterial branches showed significant differences. At the phylum level, Myxococcota, and at the genus level, *Bacillus*, *Lechevalieria*, *Streptomyces*, *Phycicoccus*, *Terrabacter*, *Nocardioides*, *Bosea*, *Roseateles*, *unclassified_f__ Comamonadaceae*, *norank_f__Roseiflexaceae*, *norank_f__BIrii41* significantly enriched in cane roots growing from TC seedlings; In contrast, *unclassified_f__Pseudonocardiaceae*, *norank_f__norank_o__norank_c__Actinobacteria*, *Mycobacterium*, *norank_f__Caulobacteraceae*, *norank_f__Micropepsaceae*, *norank_f__Magnetospirillaceae*, *Ralstonia*, *Burkholderia-Caballeronia-Paraburkholderia*, *Roseateles unclassified_f__Comamonadaceae*, *Dyella*, *unclassified_f__Rhodanobacteraceae*, *Klebsiella*, *Edaphobacter* and *Bryobacter* enriched in cane roots growing from CS seedlings.

As shown in Fig 7B, a total of 71 endophytic fungal branches showed significant differences. At the phylum level, only Ascomycota, and at the genus level, *unclassified_f__Chaetomiaceae*, *Echria*, *Fusarium*, *Myrothecium*, *unclassified_f__Clavicipitaceae*, *Monocillium*, *Acremonium*, *Sarocladium*, *Zasmidium*, *Pseudocoleophoma*, *Poaceascoma*, *Pseudorobillarda*, *Tetraplosphaeria*, *Periconia*, *Setophoma*, *Spegazzinia*, *Helicoma*, *Scytalidium*, *unclassified_o__Eurotiales*, *Aspergillus*, *unclassified_o__Trechisporales*, *unclassified_o__Sebacinales*, *Psathyrella*, *Rhizoctonia*, *Tremella*, *Cutaneotrichosporon*, *Trichosporon* and *Apiotrichum* were higher abundant in cane roots growing from TC seedlings; By contrast, at the phylum level, Chytridiomycota and unclassified_k__Fungi, and at the genus level, *Taifanglania*, *Gonytrichum*, *Paraconiothyrium*, *Deconica*, *Lysurus*, *Athelia* and *unclassified_k__Fungi* were also significantly higher abundant in cane roots growing from CS seedlings.

### Functional predictive analysis

**Fig 8:**
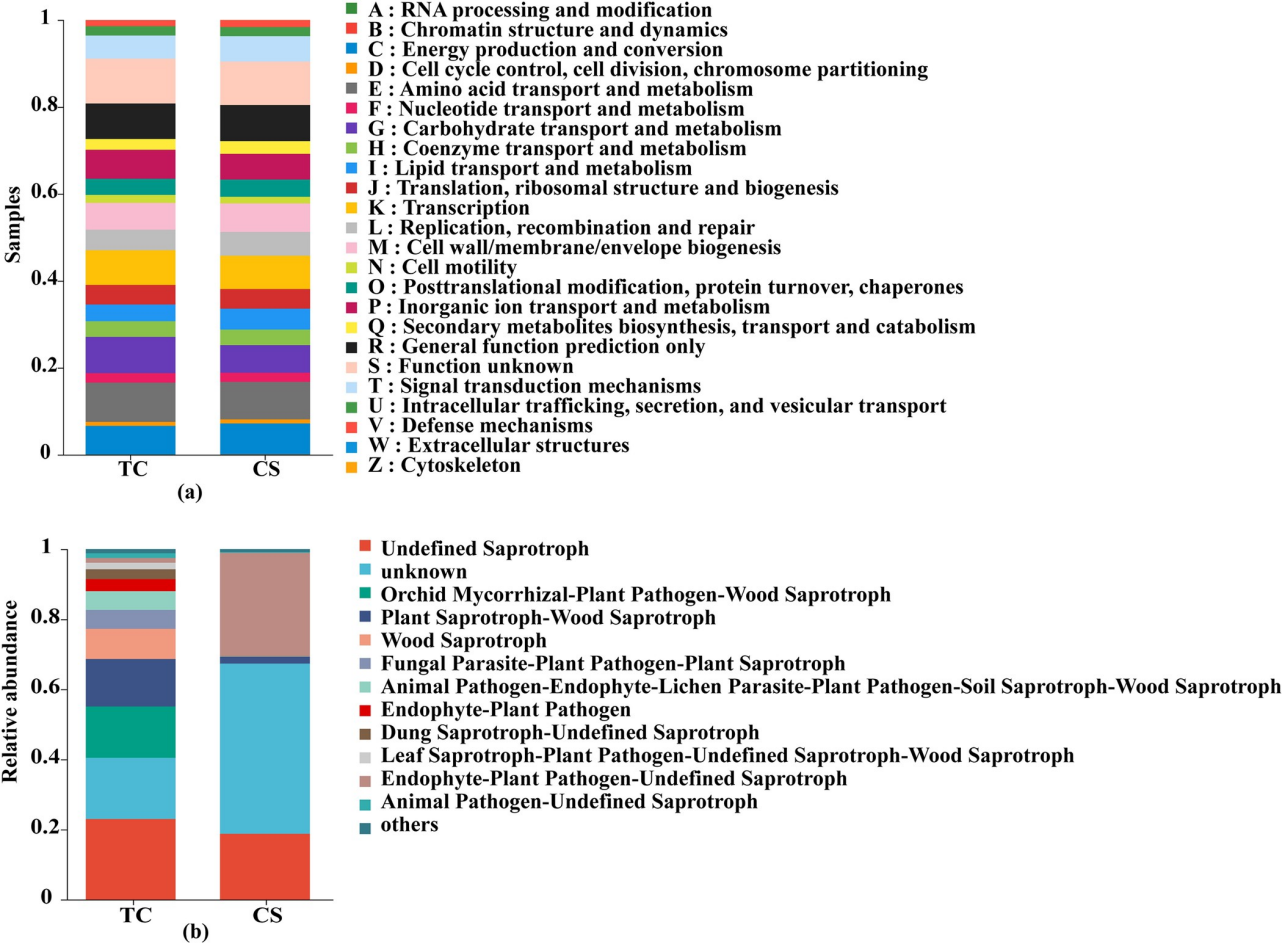
Functional prediction of bacteria and fungi in cane roots between two propagation methods. (a) bacterial PICRUSt functional prediction; (b) fungal FUNGuild functional prediction.

The functions of endophytic bacteria in cane roots growing from tissue culture and conventional seedlings were similar. And 24 functions could be found in Fig 8A. Among them, the relative abundance of carbohydrate transport and metabolism in cane roots growing from tissue culture seedlings was higher than that of conventional seedlings (CS).

In addition, the functions of endophytic fungi in cane roots were significantly different between the two propagation methods (Fig 8B). The relative abundances of endophytic fungal functions, such as Leaf Saprotroph-Plant Pathogen-Undefined Saprotroph-Wood Saprotroph, Dung Saprotroph-Undefined Saprotroph, Endophyte-Plant Pathogen, Animal Pathogen-Endophyte-Lichen Parasite-Plant Pathogen-Soil Saprotroph-Wood Saprotroph, Fungal Parasite-Plant Pathogen-Plant Saprotroph, Wood Saprotroph, Plant Saprotroph-Wood Saprotroph, Orchid Mycorrhizal-Plant Pathogen-Wood Saprotroph and Undefined Saprotroph could be detected in cane roots growing from tissue culture seedlings than those of conventional seedlings (CS).

## Discussion

The diversity of microorganisms associated with the plant root is enormous. This complex community of plant-associated microorganisms, also known as the plant’s second genome, has profound effects on host plant health and productivity [46,47]. Also, beneficial microorganisms can promote plant health by stimulating the immune system of plants [48].

Tissue culture (TC) propagation method reproduction altered the endophytic microbial community composition in cane roots which compared to those of the stem seedlings (CS). Our results showed that Chloroflexi enriched in cane roots of TC treatment. As Chloroflexi tends to live in nutrient-rich environments, which is favor in high amounts of nutrients for their growth and reproduction [49]. Meanwhile, the relative abundance of Ascomycota, was also significantly higher in tissue culture seedlings than that of CS. Previous study had confirmed that the Ascomycota could encode cellulolytic enzymes in facilitating carbon conversion processes and played a key role in nutrient cycling [29,50]. Also, most of them are typical trophozoite fungi, which they can degrade complex compounds and provide a large amount of nutrients to plants growth and other microorganisms [30,51].

Moreover, we also found that some dominant endophytic bacterial genera, such as *Pantoea*, *Bacillus*, *Streptomyces*, *Lechevalieria*, *Pseudomonas*, *Nocardioides* and *unclassified_f__Comamonadaceae* enriched in cane roots growing from TC seedlings. As previous studies had confirmed that *Pantoea*, *Bacillus*, *Streptomyces*, *Lechevalieria*, *Pseudomonas*, *Nocardioides* and *unclassified_f__Comamonadaceae* were all the nitrogen fixing bacteria in facilitating nutrient uptake by sugarcane [52–54]. Furthermore, *Pantoea* also had various functions in promoting plant growth, such as synthesis of plant hormones like IAA, cytokinins, abscisic acid and gibberellins, synthesis of iron carriers and solubilization of phosphatases [55]. Also, previous studies had confirmed that *Bacillus* possessed beneficial proerties such as iron carriers, phosphate solubilization and antifungal activity [56]. And *Bacillus* could activate protective mechanisms in plants, which included changes in cell wall structure through lignin accumulation, or production of secondary metabolites such as flavonoids, plant antitoxins, growth factors and/or thioglucosides [48,57,58]. As *Streptomyces*, *Lechevalieria* and *Nocardioides* belong to the actinomycetes family [59,60]. And Actinomycetes are well-known for the production of several antibiotics that help to improve plant health [61]. Additionally, *Unclassified_f__ Comamonadaceae* is one kind of iron autotrophic denitrifying bacteria [62]. And *Pseudomonas* produces a variety of antifungal secondary metabolites, which can protect plants from fungal infections [63].

*Codinaea* and *unclassified_c__Agaricomycetes*. *Rhizoctonia*, *Sarocladium*, *Scytalidium*, *Wongia*, *Fusarium*, *unclassified_f__Phaeosphaer*, *unclassified_c__Sordariom*, *unclassified_f__Stachybot*, *Poaceascoma*, *Microdochium*, *Arnium*, *Echria*, *Mycena* and *Exophialaare* were the special dominant endophytic fungal genera in cane roots growing from TC seedlings. Among them, *Sarocladium* has a protective effect on grass hosts against abiotic stresses and pathogens [64,65]. Also, *Fusarium* has the ability to produce anti-Fusarium compounds [66].

Further analysis based on the taxonomic level of OTUs revealed that not only the numbers of specific endophytic bacterial OTUs, but also the numbers of specific endophytic fungal OTUs increased in roots. These results suggest that more abundant endophytic microorganisms would be enriched in cane roots which produced by tissue methods. i.e., in comparison with conventional propagation method, cane health or higher stress resistance could obtained in canes by producing with tissue culture method.

## Conclusions

*Pantoea*, *Bacillus*, *Streptomyces*, *Lechevalieria*, *Pseudomonas*, *Nocardioides*, *unclassified_f__Comamonadaceae* enriched as the dominant endophytic bacterial genera, and *Rhizoctonia*, *Sarocladium*, *Scytalidium*, *Wongia*, *Fusarium*, *unclassified_f__Phaeosphaer*, *unclassified_c__Sordariom*, *unclassified_f__Stachybot*, *Poaceascoma*, *Microdochium*, *Arnium*, *Echria*, *Mycena* and *Exophiala* enriched as the dominant endophytic fungal genera in cane roots growing from the tissue culture seedlings. In contrast, *Mycobacterium*, *Massilia*, *Ralstonia*, *unclassified_f__Pseudonocardiacea*, *norank_f__Micropepsaceae*, *Leptothrix* and *Bryobacter* were the dominant endophytic bacterial genera, and *unclassified_k__Fungi*, *unclassified_f__Marasmiaceae*, *Talaromyces*, *unclassified_c__Sordariomycetes* and *Trichocladium* were the dominant endophytic fungal genera in cane roots growing from stem seedlings. Additionally, the numbers of bacterial and fungal OTUs in cane roots growing from tissue culture seedlings were all significantly higher than those of conventional seedlings. It indicates that endophytic microbial compositions in cane roots can be shaped by different propagation methods. Meanwhile, in comparison with cane conventional propagation method, cane health or higher stress resistant ability in canes can be obtained by producing with culture method.
